# Supplementary material for: Evidence of Influenza A Virus Infection in Cynomolgus Macaques, Thailand
Source: Vet Sci. 2022 Mar 13;9(3):132. doi: 10.3390/vetsci9030132 (PMC8950150; doi:10.3390/vetsci9030132)
Supplement: Supplementary file 1 [file vetsci-09-00132-s001.zip › vetsci-1604328-supplementary.pdf]

Table S1. The number of macaques with seropositive against subtypes of influenza A virus divided by demographic characteristics.

No	Place*	Province	Date of collection (2019)	Swab sample**	Serum (No)	Sex***			Age****			Influenza subtypes*****				
						M	F	NA	Sub-adult	Adult	NA	AIV H1	AIV H2	AIV H3	AIV H9	Human H1
1	Wat Khao Choeng Thain Thepharam	Chon Buri	March 25-26	B, R	25	19	6	-	8	17	-	-	-	-	-	-
2	Chom Phon Cave	Ratchaburi	March 28	NA	25	NA	NA	25	NA	NA	25	-	4	-	1	1
3	Wat Khao Somphot	Lop Buri	April 4 - 5	NA	29	16	13	-	6	23	-	-	7	-	-	-
4	Don Chao Pu Botanical Park	Amnat Charoen	April 8	O, R	24	8	16	-	NA	NA	24	-	5	-	1	-
5	Khao Ngu Stone Park	Ratchaburi	April 23	OP	24	23	1	-	3	21	-	-	3	-	-	-
6	Kosumphi Forest Park	Maha Sarakham	April 23-24	O, R	25	21	4	-	13	12	-	-	-	-	5	1
7	Wat Khao Bandai It	Phetchaburi	May 15	O, R	25	20	5	-	19	6	-	-	1	-	-	1
8	Tham Khao Luang	Phetchaburi	May 16	O, R	25	23	2	-	7	16	2	-	-	-	-	-
9	Wat Khao Thamon School	Phetchaburi	May 21	O, R	25	17	8	-	3	22	-	-	-	-	1	-
10	Wat Siri Chanthanimit Worawihan	Lop Buri	May 22	B, R	25	16	9	-	8	17	-	-	8	-	-	-
11	KaoNor-KaoKaew	Nakhon Sawan	May 27	B, R	25	20	5	-	10	15	-	1	8	-	4	-
12	Wat Khuha Sawan	Phatthalung	May 27-28	B, R	25	16	9	-	14	11	-	-	4	-	-	-
13	Wat Pa Sila Wiwek	Mukdahan	May 31	O, R	25	NA	NA	25	NA	NA	25	-	-	-	-	-
14	Mueang SAO	Chon Buri	June 4	B, R	25	22	3	-	13	12	-	8	10	2	1	-
15	Wat Khao Samo Khon	Lop Buri	June 11	B, R	26	13	13	-	6	20	-	-	5	-	-	-
16	Wat Tham Pha Sawan Thammaram	Nakhon Sawan	June 24-25	B, R	26	20	6	-	7	19	-	-	6	-	2	-
17	Khao To Phaya Wang Park	Satun	June 25	B, R	25	17	3	5	14	6	5	-	-	-	-	-
18	Mueangkhaen SAO	Si Sa Ket	June 25	NA	25	2	20	3	0	22	3	1	11	-	1	2
19	Wat Tham Phra Phothisat	Saraburi	July 9	B, R	25	17	8	-	10	15	-	-	11	-	1	1
20	Wat Tham Mongkhon Khao Changok	Phetchabun	July 20	B, R	26	20	6	-	12	14	-	-	12	-	3	-

No	Place*	Province	Date of collection (2019)	Swab sample**	Serum (No)	Sex***			Age****			Influenza subtypes*****				
						M	F	NA	Sub-adult	Adult	NA	AIV H1	AIV H2	AIV H3	AIV H9	Human H1
21	Wat Phrommawat	Chon Buri	August 1-2	O, R	25	16	9	-	16	9	-	-	2	-	-	-
22	Bang Khun Thian District	Bangkok	August 6	B, R	26	19	7	-	4	22	-	1	4	-	1	-
23	Ao Tha Lane	Krabi	August 14	B, R	25	17	8	-	11	14	-	-	-	-	-	-
24	Ko Lanta Yai	Krabi	August 27	B, R	25	18	7	-	14	11	-	-	1	-	1	-
25	Klongtamru SAO	Chon Buri	August 26-27	O, R	25	21	4	-	10	15	-	-	-	-	1	-
26	Thaksin Ratchaniwet Palace	Narathiwat	September 5-6	B, R	18	8	10	-	6	12	-	-	-	-	-	-
27	Bang Tanot SAO	Ratchaburi	November 19	O, R	23	13	10	-	19	4	-	-	-	-	-	-
				Total	672	422	192	58	233	355	84	11	102	2	23	6
											%	1.6	15.2	0.3	3.4	0.9

*: SAO, Subdistrict Administrative Organization.
**: B, buccal swab; R, rectal swab; O, oral swab; OP, Oropharyngeal swab.
***: F, Female; M, Male; NA, not available.
****: Sub-adult was defined as macaque with the age of 1-4 years, while the adult was defined as macaque with age >4 years; NA, not available.
*****: Number of individual macaques gave seropositive with HI and NT titers of ≥20. AIV, avian influenza virus; Human, human influenza virus.

Table S2. Demographic and HI/NT antibody titers of 128 individual macaques with seropositive against subtypes of influenza A virus

No	Sex*	Age**	Place***	Province	Date of collection (2019)	AIV H1N1		AIV H2N8		AIV H3N8		AIV H9N2		Human H1N1§	
						HI	NT	HI	NT	HI	NT	HI	NT	HI	NT
1	NA	NA	Chom Phon Cave	Ratchaburi	March 28	<20	ND	40	20	<20	ND	<20	ND	<20	ND
2	NA	NA	Chom Phon Cave	Ratchaburi	March 28	<20	ND	40	20	<20	ND	<20	ND	<20	ND
3	NA	NA	Chom Phon Cave	Ratchaburi	March 28	<20	ND	40	20	<20	ND	<20	ND	<20	ND
4	NA	NA	Chom Phon Cave	Ratchaburi	March 28	<20	ND	40	20	<20	ND	<20	ND	<20	ND
5	NA	NA	Chom Phon Cave	Ratchaburi	March 28	<20	ND	<20	ND	<20	ND	40	20	<20	ND
6	NA	NA	Chom Phon Cave	Ratchaburi	March 28	<20	ND	<20	ND	<20	ND	<20	ND	80	20
7	M	Adult	Wat Khao Somphot	Lop Buri	April 4-5	<20	ND	40	20	<20	ND	<20	ND	<20	ND
8	F	Adult	Wat Khao Somphot	Lop Buri	April 4-5	<20	ND	20	20	<20	ND	<20	ND	<20	ND
9	M	Sub-adult	Wat Khao Somphot	Lop Buri	April 4-5	<20	ND	40	20	<20	ND	<20	ND	<20	ND
10	M	Sub-adult	Wat Khao Somphot	Lop Buri	April 4-5	<20	ND	20	20	<20	ND	<20	ND	<20	ND
11	M	Adult	Wat Khao Somphot	Lop Buri	April 4-5	<20	ND	40	20	<20	ND	<20	ND	<20	ND
12	M	Adult	Wat Khao Somphot	Lop Buri	April 4-5	<20	ND	80	20	<20	ND	<20	ND	<20	ND
13	M	Adult	Wat Khao Somphot	Lop Buri	April 4-5	<20	ND	80	20	<20	ND	<20	ND	<20	ND
14	M	NA	Don Chao Pu Botanical Park	Amnat Charoen	April 8	<20	ND	40	20	<20	ND	<20	ND	<20	ND
15	M	NA	Don Chao Pu Botanical Park	Amnat Charoen	April 8	<20	ND	<20	ND	<20	ND	20	20	<20	ND
16	F	NA	Don Chao Pu Botanical Park	Amnat Charoen	April 8	<20	ND	40	20	<20	ND	<20	ND	<20	ND
17	M	NA	Don Chao Pu Botanical Park	Amnat Charoen	April 8	<20	ND	40	20	<20	ND	<20	ND	<20	ND
18	F	NA	Don Chao Pu Botanical Park	Amnat Charoen	April 8	<20	ND	20	20	<20	ND	<20	ND	<20	ND
19	F	NA	Don Chao Pu Botanical Park	Amnat Charoen	April 8	<20	ND	20	20	<20	ND	<20	ND	<20	ND
20	M	Adult	Kosumphi Forest Park	Maha Sarakham	April 23-24	<20	ND	<20	ND	<20	ND	<20	ND	20	20
21	M	Sub-adult	Kosumphi Forest Park	Maha Sarakham	April 23-24	<20	ND	<20	ND	<20	ND	40	20	<20	ND
22	M	Sub-adult	Kosumphi Forest Park	Maha Sarakham	April 23-24	<20	ND	<20	ND	<20	ND	40	20	<20	ND
23	F	Sub-adult	Kosumphi Forest Park	Maha Sarakham	April 23-24	<20	ND	<20	ND	<20	ND	20	20	<20	ND
24	M	Adult	Kosumphi Forest Park	Maha Sarakham	April 23-24	<20	ND	<20	ND	<20	ND	20	20	<20	ND
25	M	Adult	Kosumphi Forest Park	Maha Sarakham	April 23-24	<20	ND	<20	ND	<20	ND	20	20	<20	ND
26	F	Adult	Khao Ngu Stone Park	Ratchaburi	April 23	<20	ND	40	20	<20	ND	<20	ND	<20	ND
27	M	Adult	Khao Ngu Stone Park	Ratchaburi	April 23	<20	ND	40	20	<20	ND	<20	ND	<20	ND

No	Sex*	Age**	Place***	Province	Date of collection (2019)	AIV H1N1		AIV H2N8		AIV H3N8		AIV H9N2		Human H1N1[§]	
						HI	NT	HI	NT	HI	NT	HI	NT	HI	NT
28	M	Adult	Khao Ngu Stone Park	Ratchaburi	April 23	<20	ND	20	20	<20	ND	<20	ND	<20	ND
29	M	Sub-adult	Wat Khao Bandai It	Phetchaburi	May 15	<20	ND	<20	ND	<20	ND	<20	ND	40	20
30	M	Sub-adult	Wat Khao Bandai It	Phetchaburi	May 15	<20	ND	20	20	<20	ND	<20	ND	<20	ND
31	M	Adult	Wat Khao Thamon School	Phetchaburi	May 21	<20	ND	<20	ND	<20	ND	80	40	<20	ND
32	F	Sub-adult	Wat Siri Chanthanimit Worawihan	Lop Buri	May 22	<20	ND	40	20	<20	ND	<20	ND	<20	ND
33	F	Adult	Wat Siri Chanthanimit Worawihan	Lop Buri	May 22	<20	ND	40	20	<20	ND	<20	ND	<20	ND
34	M	Adult	Wat Siri Chanthanimit Worawihan	Lop Buri	May 22	<20	ND	20	20	<20	ND	<20	ND	<20	ND
35	F	Adult	Wat Siri Chanthanimit Worawihan	Lop Buri	May 22	<20	ND	40	20	<20	ND	<20	ND	<20	ND
36	M	Sub-adult	Wat Siri Chanthanimit Worawihan	Lop Buri	May 22	<20	ND	40	20	<20	ND	<20	ND	<20	ND
37	F	Sub-adult	Wat Siri Chanthanimit Worawihan	Lop Buri	May 22	<20	ND	40	20	<20	ND	<20	ND	<20	ND
38	F	Adult	Wat Siri Chanthanimit Worawihan	Lop Buri	May 22	<20	ND	20	20	<20	ND	<20	ND	<20	ND
39	M	Sub-adult	Wat Siri Chanthanimit Worawihan	Lop Buri	May 22	<20	ND	20	20	<20	ND	<20	ND	<20	ND
40	F	Sub-adult	Wat Khuha Sawan	Phatthalung	May 27-28	<20	ND	20	20	<20	ND	<20	ND	<20	ND
41	F	Sub-adult	Wat Khuha Sawan	Phatthalung	May 27-28	<20	ND	40	20	<20	ND	<20	ND	<20	ND
42	F	Adult	Wat Khuha Sawan	Phatthalung	May 27-28	<20	ND	20	20	<20	ND	<20	ND	<20	ND
43	M	Adult	Wat Khuha Sawan	Phatthalung	May 27-28	<20	ND	20	20	<20	ND	<20	ND	<20	ND
44	M	Adult	KaoNor-KaoKaew	Nakhon Sawan	May 27	<20	ND	40	20	<20	ND	<20	ND	<20	ND
45	M	Adult	KaoNor-KaoKaew	Nakhon Sawan	May 27	<20	ND	20	20	<20	ND	<20	ND	<20	ND
46[¶]	M	Adult	KaoNor-KaoKaew	Nakhon Sawan	May 27	20	20	40	20	<20	ND	<20	ND	<20	ND
47	M	Adult	KaoNor-KaoKaew	Nakhon Sawan	May 27	<20	ND	40	20	<20	ND	<20	ND	<20	ND
48	M	Adult	KaoNor-KaoKaew	Nakhon Sawan	May 27	<20	ND	<20	ND	<20	ND	320	160	<20	ND
49	M	Sub-adult	KaoNor-KaoKaew	Nakhon Sawan	May 27	<20	ND	<20	ND	<20	ND	20	20	<20	ND
50	M	Adult	KaoNor-KaoKaew	Nakhon Sawan	May 27	<20	ND	<20	ND	<20	ND	40	20	<20	ND
51	M	Adult	KaoNor-KaoKaew	Nakhon Sawan	May 27	<20	ND	40	20	<20	ND	<20	ND	<20	ND
52	M	Sub-adult	KaoNor-KaoKaew	Nakhon Sawan	May 27	<20	ND	20	20	<20	ND	<20	ND	<20	ND
53	F	Sub-adult	KaoNor-KaoKaew	Nakhon Sawan	May 27	<20	ND	40	20	<20	ND	<20	ND	<20	ND
54	F	Sub-adult	KaoNor-KaoKaew	Nakhon Sawan	May 27	<20	ND	40	20	<20	ND	<20	ND	<20	ND
55	F	Sub-adult	KaoNor-KaoKaew	Nakhon Sawan	May 27	<20	ND	<20	ND	<20	ND	20	20	<20	ND
56	M	Sub-adult	Mueang SAO	Chon Buri	June 4	20	20	<20	ND	<20	ND	<20	ND	<20	ND
57	M	Sub-adult	Mueang SAO	Chon Buri	June 4	<20	ND	20	20	<20	ND	<20	ND	<20	ND
58[¶]	M	Adult	Mueang SAO	Chon Buri	June 4	20	20	80	20	<20	ND	<20	ND	<20	ND
59	M	Adult	Mueang SAO	Chon Buri	June 4	<20	ND	40	20	<20	ND	<20	ND	<20	ND

No	Sex*	Age**	Place***	Province	Date of collection (2019)	AIV H1N1		AIV H2N8		AIV H3N8		AIV H9N2		Human H1N1§	
						HI	NT	HI	NT	HI	NT	HI	NT	HI	NT
60	M	Sub-adult	Mueang SAO	Chon Buri	June 4	<20	ND	20	20	<20	ND	<20	ND	<20	ND
61¶	M	Adult	Mueang SAO	Chon Buri	June 4	<20	ND	40	20	<20	ND	20	20	<20	ND
62¶	F	Sub-adult	Mueang SAO	Chon Buri	June 4	20	40	40	20	<20	ND	<20	ND	<20	ND
63	M	Adult	Mueang SAO	Chon Buri	June 4	<20	ND	20	20	<20	ND	<20	ND	<20	ND
64	M	Adult	Mueang SAO	Chon Buri	June 4	<20	ND	20	20	<20	ND	<20	ND	<20	ND
65¶	M	Adult	Mueang SAO	Chon Buri	June 4	20	40	40	20	<20	ND	<20	ND	<20	ND
66¶	F	Sub-adult	Mueang SAO	Chon Buri	June 4	40	20	<20	ND	40	20	<20	ND	<20	ND
67	M	Adult	Mueang SAO	Chon Buri	June 4	20	20	<20	ND	<20	ND	<20	ND	<20	ND
68	F	Sub-adult	Mueang SAO	Chon Buri	June 4	20	20	<20	ND	<20	ND	<20	ND	<20	ND
69#	M	Sub-adult	Mueang SAO	Chon Buri	June 4	20	20	40	20	20	20	<20	ND	<20	ND
70	F	Adult	Wat Khao Samo Khon	Lop Buri	June 11	<20	ND	20	20	<20	ND	<20	ND	<20	ND
71	F	Adult	Wat Khao Samo Khon	Lop Buri	June 11	<20	ND	20	20	<20	ND	<20	ND	<20	ND
72	F	Adult	Wat Khao Samo Khon	Lop Buri	June 11	<20	ND	20	20	<20	ND	<20	ND	<20	ND
73	F	Sub-adult	Wat Khao Samo Khon	Lop Buri	June 11	<20	ND	20	20	<20	ND	<20	ND	<20	ND
74	F	Adult	Wat Khao Samo Khon	Lop Buri	June 11	<20	ND	20	20	<20	ND	<20	ND	<20	ND
75	M	Adult	Wat Tham Pha Sawan Thammaram	Nakhon Sawan	June 24-25	<20	ND	<20	ND	<20	ND	20	20	<20	ND
76	M	Adult	Wat Tham Pha Sawan Thammaram	Nakhon Sawan	June 24-25	<20	ND	40	20	<20	ND	<20	ND	<20	ND
77	M	Adult	Wat Tham Pha Sawan Thammaram	Nakhon Sawan	June 24-25	<20	ND	20	20	<20	ND	<20	ND	<20	ND
78	M	Adult	Wat Tham Pha Sawan Thammaram	Nakhon Sawan	June 24-25	<20	ND	40	20	<20	ND	<20	ND	<20	ND
79	M	Adult	Wat Tham Pha Sawan Thammaram	Nakhon Sawan	June 24-25	<20	ND	20	20	<20	ND	<20	ND	<20	ND
80	F	Sub-adult	Wat Tham Pha Sawan Thammaram	Nakhon Sawan	June 24-25	<20	ND	<20	ND	<20	ND	20	20	<20	ND
81	F	Sub-adult	Wat Tham Pha Sawan Thammaram	Nakhon Sawan	June 24-25	<20	ND	40	20	<20	ND	<20	ND	<20	ND
82	F	Sub-adult	Wat Tham Pha Sawan Thammaram	Nakhon Sawan	June 24-25	<20	ND	20	20	<20	ND	<20	ND	<20	ND
83	F	Adult	Mueangkhaen SAO	Si Sa Ket	June 25	<20	ND	20	20	<20	ND	<20	ND	<20	ND
84	F	Adult	Mueangkhaen SAO	Si Sa Ket	June 25	<20	ND	40	20	<20	ND	<20	ND	<20	ND
85¶	F	Adult	Mueangkhaen SAO	Si Sa Ket	June 25	<20	ND	20	20	<20	ND	<20	ND	40	20
86	F	Adult	Mueangkhaen SAO	Si Sa Ket	June 25	<20	ND	20	20	<20	ND	<20	ND	<20	ND
87¶	F	Adult	Mueangkhaen SAO	Si Sa Ket	June 25	<20	ND	40	20	<20	ND	<20	ND	40	20
88	F	Adult	Mueangkhaen SAO	Si Sa Ket	June 25	<20	ND	40	20	<20	ND	<20	ND	<20	ND
89	F	Adult	Mueangkhaen SAO	Si Sa Ket	June 25	<20	ND	40	20	<20	ND	<20	ND	<20	ND
90¶	F	Adult	Mueangkhaen SAO	Si Sa Ket	June 25	20	20	80	20	<20	ND	<20	ND	<20	ND
91	F	Adult	Mueangkhaen SAO	Si Sa Ket	June 25	<20	ND	40	20	<20	ND	<20	ND	<20	ND

No	Sex*	Age**	Place***	Province	Date of collection (2019)	AIV H1N1		AIV H2N8		AIV H3N8		AIV H9N2		Human H1N1§	
						HI	NT	HI	NT	HI	NT	HI	NT	HI	NT
92	F	Adult	Mueangkhaen SAO	Si Sa Ket	June 25	<20	ND	80	20	<20	ND	<20	ND	<20	ND
93	NA	NA	Mueangkhaen SAO	Si Sa Ket	June 25	<20	ND	<20	ND	<20	ND	80	20	<20	ND
94	NA	NA	Mueangkhaen SAO	Si Sa Ket	June 25	<20	ND	40	20	<20	ND	<20	ND	<20	ND
95	M	Sub-adult	Wat Tham Phra Phothisat	Saraburi	July 9	<20	ND	20	20	<20	ND	<20	ND	<20	ND
96¶	M	Adult	Wat Tham Phra Phothisat	Saraburi	July 9	<20	ND	20	20	<20	ND	<20	ND	20	20
97	F	Sub-adult	Wat Tham Phra Phothisat	Saraburi	July 9	<20	ND	40	20	<20	ND	<20	ND	<20	ND
98	M	Sub-adult	Wat Tham Phra Phothisat	Saraburi	July 9	<20	ND	20	20	<20	ND	<20	ND	<20	ND
99	F	Adult	Wat Tham Phra Phothisat	Saraburi	July 9	<20	ND	20	20	<20	ND	<20	ND	<20	ND
100	M	Sub-adult	Wat Tham Phra Phothisat	Saraburi	July 9	<20	ND	<20	ND	<20	ND	20	20	<20	ND
101	M	Sub-adult	Wat Tham Phra Phothisat	Saraburi	July 9	<20	ND	80	20	<20	ND	<20	ND	<20	ND
102	F	Sub-adult	Wat Tham Phra Phothisat	Saraburi	July 9	<20	ND	20	20	<20	ND	<20	ND	<20	ND
103	F	Sub-adult	Wat Tham Phra Phothisat	Saraburi	July 9	<20	ND	40	20	<20	ND	<20	ND	<20	ND
104	F	Sub-adult	Wat Tham Phra Phothisat	Saraburi	July 9	<20	ND	20	20	<20	ND	<20	ND	<20	ND
105	M	Adult	Wat Tham Phra Phothisat	Saraburi	July 9	<20	ND	20	20	<20	ND	<20	ND	<20	ND
106	M	Adult	Wat Tham Phra Phothisat	Saraburi	July 9	<20	ND	20	20	<20	ND	<20	ND	<20	ND
107	M	Adult	Wat Tham Mongkhon Khao Changok	Phetchabun	July 20	<20	ND	40	20	<20	ND	<20	ND	<20	ND
108¶	F	Adult	Wat Tham Mongkhon Khao Changok	Phetchabun	July 20	<20	ND	40	20	<20	ND	20	20	<20	ND
109	F	Adult	Wat Tham Mongkhon Khao Changok	Phetchabun	July 20	<20	ND	40	20	<20	ND	<20	ND	<20	ND
110	M	Sub-adult	Wat Tham Mongkhon Khao Changok	Phetchabun	July 20	<20	ND	40	20	<20	ND	<20	ND	<20	ND
111	M	Sub-adult	Wat Tham Mongkhon Khao Changok	Phetchabun	July 20	<20	ND	40	20	<20	ND	<20	ND	<20	ND
112	M	Adult	Wat Tham Mongkhon Khao Changok	Phetchabun	July 20	<20	ND	80	20	<20	ND	<20	ND	<20	ND
113	M	Sub-adult	Wat Tham Mongkhon Khao Changok	Phetchabun	July 20	<20	ND	20	20	<20	ND	<20	ND	<20	ND
114	F	Sub-adult	Wat Tham Mongkhon Khao Changok	Phetchabun	July 20	<20	ND	20	20	<20	ND	<20	ND	<20	ND
115	M	Adult	Wat Tham Mongkhon Khao Changok	Phetchabun	July 20	<20	ND	40	20	<20	ND	<20	ND	<20	ND
116¶	F	Adult	Wat Tham Mongkhon Khao Changok	Phetchabun	July 20	<20	ND	20	20	<20	ND	20	20	<20	ND
117¶	F	Sub-adult	Wat Tham Mongkhon Khao Changok	Phetchabun	July 20	<20	ND	20	20	<20	ND	20	20	<20	ND
118	M	Sub-adult	Wat Tham Mongkhon Khao Changok	Phetchabun	July 20	<20	ND	40	20	<20	ND	<20	ND	<20	ND
119	F	Adult	Bang Khun Thian District	Bangkok	August 6	<20	ND	40	20	<20	ND	<20	ND	<20	ND

No	Sex*	Age**	Place***	Province	Date of collection (2019)	AIV H1N1		AIV H2N8		AIV H3N8		AIV H9N2		Human H1N1§	
						HI	NT	HI	NT	HI	NT	HI	NT	HI	NT
120	M	Adult	Bang Khun Thian District	Bangkok	August 6	<20	ND	40	20	<20	ND	<20	ND	<20	ND
121	M	Sub-adult	Bang Khun Thian District	Bangkok	August 6	<20	ND	20	20	<20	ND	<20	ND	<20	ND
122¶	F	Adult	Bang Khun Thian District	Bangkok	August 6	40	20	20	20	<20	ND	<20	ND	<20	ND
123	M	Adult	Bang Khun Thian District	Bangkok	August 6	<20	ND	<20	ND	<20	ND	20	20	<20	ND
124	F	Sub-adult	Wat Phrommawat	Chon Buri	August 1-2	<20	ND	40	20	<20	ND	<20	ND	<20	ND
125	F	Sub-adult	Wat Phrommawat	Chon Buri	August 1-2	<20	ND	40	20	<20	ND	<20	ND	<20	ND
126	M	Adult	Ko Lanta Yai	Krabi	August 27	<20	ND	<20	ND	<20	ND	20	20	<20	ND
127	F	Sub-adult	Ko Lanta Yai	Krabi	August 27	<20	ND	40	20	<20	ND	<20	ND	<20	ND
128	F	Adult	Klongtamru SAO	Chon Buri	August 26-27	<20	ND	<20	ND	<20	ND	20	20	<20	ND
					Total	11		102		2		23		6	

*: F, Female; M, Male; NA, not available.

**: Sub-adult was defined as macaque with the age of 1-4 years, while the adult was defined as macaque with age >4 years; NA, not available.

***: SAO, Subdistrict Administrative Organization.

§: AIV, avian influenza virus; Human, human influenza virus; HI, hemagglutination inhibition antibody titer; NT, neutralizing antibody titer; ND, not done. Both HI and NT titer of ≥20 were considered as seropositive. Only HI titer ≥20 was further determined for NT titer.

¶: Individual macaque gave seropositive results for both virus subtypes.

#: Individual macaque gave seropositive results for three virus subtypes.
